# Radiological approaches to COVID-19 pneumonia

**DOI:** 10.3906/sag-2004-332

**Published:** 2020-08-26

**Authors:** Furkan UFUK, Recep SAVAŞ, Tuncay HAZIROLAN

**Affiliations:** 1 Department of Radiology, Faculty of Medicine, Pamukkale University, Denizli Turkey; 2 Department of Radiology, Faculty of Medicine, Ege University, İzmir Turkey; 3 Department of Radiology, Faculty of Medicine, Hacettepe University, Ankara Turkey

**To the Editor,**

We read with great interest the article titled “Radiological approaches to COVID-19 pneumonia” by Akçay et al. in the latest issue of the Turkish Journal of Medical Sciences [1]. The authors provided a review of the radiological findings of novel coronavirus disease (COVID-19) pneumonia. We would like to highlight some missing radiological findings in this article, and we want to contribute to some critical radiological findings.

First, the authors stated that unenhanced chest computed tomography (CT) should be considered for early diagnosis of viral disease in suspected patients with a normal chest x-ray [1]. However, the Fleischner Society does not recommend chest CT in patients with suspected COVID-19 and mild clinical features unless they are at risk for disease progression or worsening respiratory status [2]. It should not be forgotten that chest CT may be entirely normal in an essential part of the patients with COVID-19 and CT findings in patients with COVID-19 pneumonia are often nonspecific, as they are often found in other viral causes of pneumonia, fungal infections, and organizing pneumonia [3]. For these reasons, we suggest that in patients with a suspicion of COVID-19, the CT indication should be limited to the expected benefit that can lead to a change in the treatment or management of the patient.

Second, the patient in the first image was presented as an atypical appearance for COVID-19 pneumonia, but CT shows round-shaped, peripheral ground-glass opacities (GGO) in both lower lobes [1]. However, in the expert consensus statement on reporting chest CT findings related to COVID-19 classifications, these findings are classified as “typical” [4]. Therefore, CT images are typical for COVID-19 pneumonia. Moreover, the authors interpret Figure 6 with the statement that “unilateral multilobar focal consolidation areas are seen on CT images”. However, when the CT images are evaluated, bilateral, multilobar ground-glass opacities (GGO), and mixed attenuation (GGO with consolidation) areas are observed.

Third, some critical chest CT findings such as crazy paving pattern, reversed halo sign, air bronchogram sign, and subsegmental vascular enlargement inside or around the lesion are common in patients with COVID-19 pneumonia. These were not mentioned in detail or were only included in the tables [1]. Among these findings, vascular enlargement is an interesting chest CT feature described as subsegmental vascular widening with a diameter of >3 mm inside or around the opacities, which was reported in 59% to 89% of COVID-19 patients [5,6]. Although the cause of vascular enlargement is not fully understood, it is suggested that it may be due to proinflammatory factors [6].

Fourth, chest CT is not just a diagnostic or screening method in patients with suspected COVID-19. Chest CT is especially crucial in determining the severity of the disease and ensuring patient triage. The severity of pneumonia in CT has been shown to predict the intensive care unit indication, the need for mechanical ventilation, and the patient’s prognosis [4,7]. In patients with COVID-19 pneumonia, visual (semiquantitative) and quantitative analysis methods have been described for the estimating the severity of clinical disease [7,8]. Therefore, the percentage of pulmonary involvement must be specified during CT evaluation.

## References

[ref1] (2020). Radiological approaches to COVID-19 pneumonia. Turkish Journal of Medical Sciences.

[ref2] (2020). The role of chest imaging in patient management during the COVID-19 pandemic: a multinational consensus statement from the Fleischner Society. Chest.

[ref3] (2020). Relation between chest CT findings and clinical conditions of coronavirus disease (COVID-19) pneumonia: a multicenter study. American Journal of Roentgenology.

[ref4] (2020). Radiological Society of North America expert consensus statement on reporting chest CT findings related to COVID-19. Endorsed by the Society of Thoracic Radiology, the American College of Radiology, and RSNA. Radiology: Cardiothoracic Imaging.

[ref5] (2020). Coronavirus Disease 2019 (COVID-19): Role of chest CT in diagnosis and management. American Journal of Roentgenology.

[ref6] (2020). Chest CT features of COVID-. Radiology.

[ref7] (2020). Well-aerated lung on admitting chest CT to predict adverse outcome in COVID-19 pneumonia. Radiology.

[ref8] (2020). CT image visual quantitative evaluation and clinical classification of coronavirus disease (COVID-19). European Radiology.

